# Efficacy of laparoscopic repeat hepatectomy compared with open repeat hepatectomy: a single-center, propensity score matching study

**DOI:** 10.1186/s12957-022-02668-1

**Published:** 2022-06-13

**Authors:** Zefeng Shen, Jingwei Cai, Jiaqi Gao, Junhao Zheng, Liye Tao, Yuelong Liang, Junjie Xu, Xiao Liang

**Affiliations:** Department of General Surgery, Sir Run Run Shaw Hospital, College of Medicine, Zhejiang University, Hangzhou, Zhejiang Province China

**Keywords:** Laparoscopy, Hepatocellular cancer, Hepatectomy, Recurrence

## Abstract

**Introduction:**

Laparoscopic repeat hepatectomy (LRH) is considered to be a technically challenging procedure which has not been widely applied. This study aimed to assess the accessibility and security of LRH for patients with hepatic tumor recurrence.

**Methods:**

Between January 2010 and October 2020, we performed 48 LRHs and 31 open repeat hepatectomies (ORHs) for recurrent liver cancer. LRHs were matched to ORHs (1:1) using propensity score matching (PSM) created by comparing preoperative factors. The perioperative data of patients were retrospectively analyzed, including baseline data, operative time, intraoperative blood loss, pathology, days of postoperative stay, complication morbidity, and mortality within 30 days. Overall survival and recurrence-free survival rates with appropriate follow-up were obtained to evaluate the long-term outcomes.

**Results:**

Compared with the ORH, LRH was related with shorter operative duration (169.9 versus 232.9 ml, *p* < 0.01), less intraoperative bleeding (100.0 versus 500.0 ml, *p* < 0.01), lower rate of blood transfusion (8.3% versus 58.1%, *p* < 0.01), and shorter hospitalization (5.0 versus 11.0 days, *p* < 0.01). The median follow-up was 31 months. The LRH 1-, 3-, and 5-year overall survival were 77.1%, 61.6%, and 46.2% versus 82.3%, 66.5%, and 29.5% for ORH (*p* = 0.77). The 1-, 3-, and 5-year disease-free survival rates of the two groups were 73.4%, 62.0%, and 44.3% versus 66.1%, 44.1%, and 14.7%, respectively (*p* = 0.22).

**Conclusions:**

Laparoscopic repeated hepatectomy is safe and practicable with great short-term results for selected patients.

## Introduction

Hepatectomy has been considered as one of the most effective treatments for primary liver cancer (PLC) and secondary liver malignancies. And the indications for hepatic resection have been broadened with the development of laparoscopic instruments and techniques [1, 2]. Consequently, laparoscopic hepatectomy (LH) rather than open hepatectomy (OH) is deemed as the preferred option in various situation. While confronted with secondary liver malignancies, surgeons become hesitant to choose LH in consideration of the progression of liver cirrhosis, the presence of postoperative adhesion, and the anatomic structure changes caused by the previous hepatectomy [3]. So far, there have been limited reports concerning the role of laparoscopic repeat hepatectomy (LRH) [4]. Previous research has discovered that repeat hepatectomy (RH) remains an efficacious treatment for hepatocellular carcinoma (HCC) recurrence and colorectal liver metastasis (CRLM), often accomplished by the open approach despite its severe surgical trauma [5–8]. It is reported that the prognosis of patients after surgery is often affected by frequent tumor recurrence, with high recurrence rates within 5 years after surgery being respectively 50–70% and 60% in HCC and CRLM cases [9, 10], and it is of urgent significance to explore the modified forms of current repeat hepatectomy. Therefore, the objective of our research was to investigate the efficacy of laparoscopic repeat hepatectomy (LRH) for HCC recurrence and CRLM in comparison with open repeat hepatectomy (ORH).

## Materials and methods

Between January 2010 to October 2020, 79 consecutive patients underwent repeat hepatectomy in the Department of General Surgery, Sir Run Run Shaw Hospital. The inclusion criteria were as per the following:

1. Recurrent HCC, CRLM, or other hepatic neoplasm
2. A well-compensated liver function (Child-Pugh classes A or B) without severe portal hypertension
3. No major vessel or bile duct invasion or metastasis

Exclusion criteria were as per the following:

1. Patients with identified extrahepatic metastasis
2. Surgery only for laparoscopic exploration
3. The tumor invaded the major vessels.
4. Patients with poor cardiopulmonary function and will not tolerate the surgery

According to whether the second hepatectomy was performed under laparotomy or laparoscopy, patients were separated into LRH and ORH groups. Morbidity was classified as indicated by Clavien classification [11]. In the present study, we compared the preoperative, intraoperative, and postoperative data of the LRH group with those of the ORH group, including sex, age, body mass index (BMI), HBV infection, cirrhosis, Child–Pugh score system, American Society of Anesthesiologists (ASA) classification, preoperative laboratory results, number of tumor lesions, tumor size, tumor localization, pathological diagnosis, operative time, blood loss, Pringle maneuver, type of resection, the postoperative complications, and the length of the postoperative hospital stay. We performed 1:1 propensity score matching with a 0.05 caliper between the groups to minimize selection biases in the demographic characteristics and tumor characteristics. Propensity scores were calculated in R version 3.6.2 using a logistic regression model. Age, BMI, HBV status, and tumor size were enrolled in the model. Follow-up was accomplished in HCC patients by trained staff every 3 months postoperatively for tumor surveillance in the initial 2 years after discharge, and afterward at half-year spans, thereafter.

Our study was permitted by the hospital ethical committee of the Sir Run Run Shaw Hospital (Hangzhou, China), following the principles of the Declaration of Helsinki and its appendices [12].

All statistical analyses were performed using SPSS 23.0 (IBM Corp., Armonk, NY, USA). Kolmogorov–Smirnov normal distribution testing was performed on all continuous variables. Continuous data with normal distribution were presented as mean and standard deviation (XS), and differences for independent groups were analyzed by Student’s *t*-test. Non-normally distributed data were described as median with interquartile range (IQR), and the Mann–Whitney *U*-test was employed to evaluate differences between the groups. Categorical variables were expressed as absolute numbers with percentages and were compared between the groups using the chi-squared test. Survival analysis was performed using the Kaplan–Meier method and log-rank test. All comparisons with a two-sided nominal *p* < 0.05 were considered to indicate statistical significance.

### Surgical approaches

All patients underwent general anesthesia and intubation. Patients were placed in a right lateral decubitus position for lesions in the right lobe. The first trocar was carefully embedded near the umbilicus and kept away from the original incision. Laparoscopic exploration was performed after establishing pneumoperitoneum, which was maintained at 8–12 mmHg. When the second trocar was inserted, dissection of intra-abdominal adhesions began. Combined with the “triangular principle” of laparoscopic surgery, three or four additional trocars could be placed to ensure exposure of the operative area and ease of operation. There was no need to dissect all adhesions under flexible laparoscopy unless the adhesions obscured the operative field. Moreover, adhesion bands could be stretched under the magnifying field of a modern laparoscope, with pneumoperitoneum, to achieve accurate adhesion dissection [13]. Most adhesions in front of the hepatic hilum were safely divided using an ultrasonic scalpel. Pringle’s maneuver was not routinely performed, and selective hemi-hepatic vascular occlusion was conventionally applied in hemi-hepatectomy. Liver parenchyma transection was performed using a laparoscopic Peng’s multifunctional operative dissector (LPMOD) or a harmonic scalpel.

ORH was applied via a “reverse L-shaped,” midline incision or subcostal incision. Hepatic resection was performed by the harmonic scalpel. Pringle’s maneuver was not performed in some patients with severe adhesions surrounding the hepatoduodenal ligament.

## Results

During the investigation time frame, 79 consecutive patients received repeat hepatectomy, comprising 48 LRHs and 31 ORHs. One patient undergoing LRH was converted to laparotomy owing to tumor invasion of the hepatic common duct. Two cases of LRH were converted to laparotomy owing to postoperative severe adhesions and difficulty exposing the tumor intraoperatively, respectively.

### Baseline variables and outcomes before matching

The baseline demographics before matching are summed up in Table 1. The most noticeable difference between the two groups was that the percentage of patients with HBV in the ORH group was more than that in the LRH group (87.1% versus 62.5%, respectively; *p* < 0.01). Large lesions were more frequent (2.3 cm versus 4.2 cm; *p* < 0.01) in the LRH vs ORH group, respectively (Table 2). However, no significant intergroup differences were observed for the proportion of patients with tumors located in segment VII, VIII, or I (25.0% in the ORH group and 29.0% in the LRH group; *p* = 0.70). Thirty-six patients of LRH group and 26 patients of ORH group were diagnosed with HCC, pathologically.

**Table 1:** Study population and baseline clinical characteristics before and after PSM

	Before propensity score matching			After propensity score matching		
	LRH (*n* = 48)	ORH (*n* = 31)	*P*-value	LRH (*n* = 17)	ORH (*n* = 17)	*P*-value
Gender ratio (M:F)	43:5	28:3	0.92	16:1	16:1	1.0
Age, years	63 (55−68)	61 (57−68)	0.25	62.2 ± 9.8	61.3 ± 9.6	0.83
BMI	22.5 ± 3.3	23.8 ± 3.2	0.08	23.2 ± 4.1	23.4 ± 3.6	0.90
HBV	30 (62.5)	27 (87.1)	0.02	12 (70.6)	13 (76.5)	0.70
Liver cirrhosis	22 (45.8)	19 (61.3)	0.18	8 (47.1)	9 (52.9)	0.73
Previous hepatectomy			0.11			0.09
Laparoscopic	29 (62.5)	13 (41.9)		11 (64.7)	6 (35.3)	
Open	19 (37.5)	18 (58.1)		6 (35.3)	11 (64.7)	
Child–Pugh grade			0.85			0.63
A	41 (85.4)	26 (83.9)		14 (82.4)	15 (88.2)	
B	7 (14.6)	5 (16.1)		3 (17.6)	2 (11.8)	
ASA score			0.19			0.70
II	40 (83.3)	22 (71.0)		13 (76.5)	12 (70.6)	
III	8 (16.7)	9 (29.0)		4 (23.5)	5 (29.4)	
Preoperative laboratory results						
TBil in mg/dL	13.7 (9.8−18.6)	14.3 (9.7−21.5)	0.93	15.0 ± 6.7	13.1 ± 6.3	0.44
Alb in g/L	40.3 ± 4.3	39.1 ± 6.3	0.31	40.0 ± 3.9	40.2 ± 5.4	0.89
PT in seconds	13.4 (13.0−15.2)	13.8 (13.1−14.6)	0.58	14.0 ± 1.5	13.7 ± 1.5	0.63
PLT/mm^3^ × 10^3^	124.0 (90.3−146.8)	129.0 (87.0−158.0)	0.93	130.1 ± 52.3	145.7 ± 59.2	0.60

*BMI* body mass index, *TBil* total bilirubin, *Alb* albumin, *PT* prothrombin time, *PLT* platelet, *PSM* propensity score matching, *LRH* laparoscopic repeat hepatectomy, *ORH* open repeat hepatectomy, *ASA* American Society of Anesthesiologists, *M* male, *F* female, *HBV* hepatitis virus B

**Table 2:** Tumor characteristics and operative outcomes before and after PSM

	Before propensity score matching			After propensity score matching		
	LRH (*n* = 48)	ORH (*n* = 31)	*P*-value	LRH (*n* = 17)	ORH (*n* = 17)	*P*-value
Tumor size (cm)	2.3 ± 1.0	4.2 ± 2.7	< 0.01	2.9 ± 0.9	2.8 ± 1.4	0.79
Number of tumors			0.13			0.67
Single	42 (87.5)	23 (74.2)		14 (82.4)	13 (76.5)	
Multiple	6 (12.5)	8 (25.8)		3 (17.6)	4 (23.5)	
Location (Couinaud section)			0.70			0.23
Segments II, III, IV, V, VI	32 (66.7)	18 (58.1)		11 (64.7)	6 (35.3)	
Segments VII, VIII, I	12 (25.0)	9 (29.0)		5 (29.4)	9 (52.9)	
Bilober	4 (8.3)	4 (12.9)		1 (5.9)	2 (11.8)	
Pathological diagnosis			-			-
HCC	36 (75.0)	26 (83.9)		13 (76.5)	14 (82.4)	
ICC	3 (6.3)	2 (6.5)		1 (5.9)	1 (5.9)	
CRLM	8 (16.6)	2 (6.5)		3 (17.6)	2 (11.8)	
Hepatic benign tumor	1 (2.1)	1 (3.2)		0	0	

*PSM* propensity score matching, *LRH* laparoscopic repeat hepatectomy, *ORH* open repeat hepatectomy, *HCC* hepatocellular carcinoma, *ICC* intrahepatic cholangiocarcinoma, *CRLM* colorectal liver metastases

The patients’ perioperative outcomes are shown in Table 3. More operative duration was recorded in the ORH group (232.9 min versus 169.9 min, respectively; *p* < 0.01). The median estimated intraoperative blood loss in the LRH group was significantly reduced than that in the ORH group (100 ml versus 500 ml, respectively; *p* < 0.01), and more patients of ORH group received blood transfusions intraoperatively compared with the LRH group (58.1% versus 8.3%, respectively; *p* < 0.01). Patients in both groups underwent R0 resection, and there were no differences with regard to complications after operation. During the immediate postoperative period, 3 patients in the LRH group and 13 patients in the ORH group developed severe postoperative complications (Clavien–Dindo grade > III). Two patients died from liver failure within 30 days after the primary operation. Shorter postoperative hospital stay was observed in the LRH group than the ORH group (5.0 days versus 13.0 days, respectively; *p* < 0.01).

**Table 3:** Intraoperative and postoperative outcomes before and after PSM

	LRH (*n* = 48)	ORH (*n* = 31)	*P*-value	LRH (*n* = 17)	ORH (*n* = 17)	*P*-value
Operative time (min)	169.9 ± 81.5	232.9 ± 83.1	< 0.01	212.9 ± 91.3	226.9 ± 70.5	0.94
Blood loss (ml)	100.0 (50.0–112.5)	500.0 (250.0–1000.0)	< 0.01	100.0 (100.0–200.0)	500.0 (250.0–800.0)	< 0.01
Pringle maneuver	14 (29.2)	17 (54.8)	0.13	3 (17.6)	6 (35.3)	0.70
Transfusion	4 (8.3)	18 (58.1)	< 0.01	4 (23.5)	10 (58.8)	0.04
Type of resection			0.07			0.47
Major resection	11 (22.9)	13 (41.9)		5 (29.4)	7 (41.2)	
Minor resection	37 (77.1)	18 (58.1)		12 (70.6)	10 (58.8)	
R0 resection rate	100%	100%	-	100%	100%	-
Conversion	3 (6.3)	-		1 (5.8)	-	
30-day mortality	0	2 (6.5)	-	0	1 (5.9)	-
Complications (Clavien–Dindo)	10 (20.8)	16 (54.2)	0.07	6 (35.3)	10 (58.8)	0.02
I	2 (4.2)	2 (6.5)		2 (11.8)	2 (11.8)	
II	5 (10.4)	1 (3.2)		4 (23.5)	0	
III	2 (4.2)	5 (16.1)		0	4 (23.5)	
IV	1 (2.1)	6 (19.4)		0	2 (11.8)	
V	0	2 (6.5)		0	2 (11.8)	
Postoperative hospital day	5.0 (4.0–8.0)	13. 0 (9.0–19.0)	< 0.01	7.0 (4.0–10.0)	11.0 (9.0–20.0)	0.01

*PSM* propensity score matching, *LRH* laparoscopic repeat hepatectomy, *ORH* open repeat hepatectomy

### Baseline variables and outcomes after matching

**Fig. 1:**
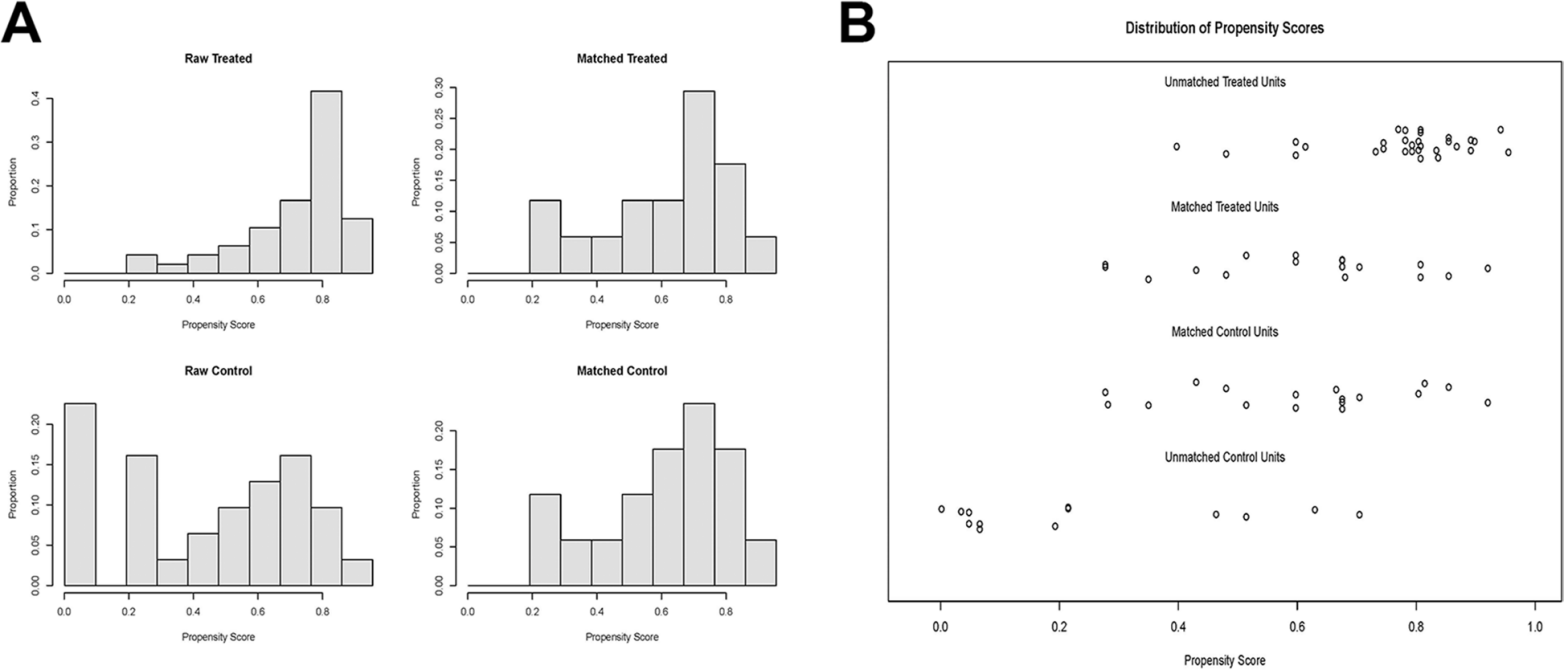
**A** Hist plot of the propensity score before and after PSM. **B** Propensity score matching jitter plot

After propensity score matching, 17 LRHs were matched with 17 ORHs, and the details of the PSM were displayed as the hist plot and jitter plot in Fig. 1. Demographic and clinical characteristics were comparable, without any significant difference between the groups (Table 1). Higher intraoperative blood loss was observed in the ORH group compared with the LRH group (500 ml versus 100 ml, respectively; *p* < 0.01); blood transfusion requirement was significantly lower in the LRH group (23.5% versus 58.8%, respectively; *p* = 0.03); and postoperative morbidity and mortality rates in the ORH group were higher compared with the LRH group. Moreover, LRH was associated with shorter hospitalization vs ORH (7.0 days versus 11.0 days, respectively; *p* = 0.01).

### Long-term postoperative outcomes

**Fig. 2:**
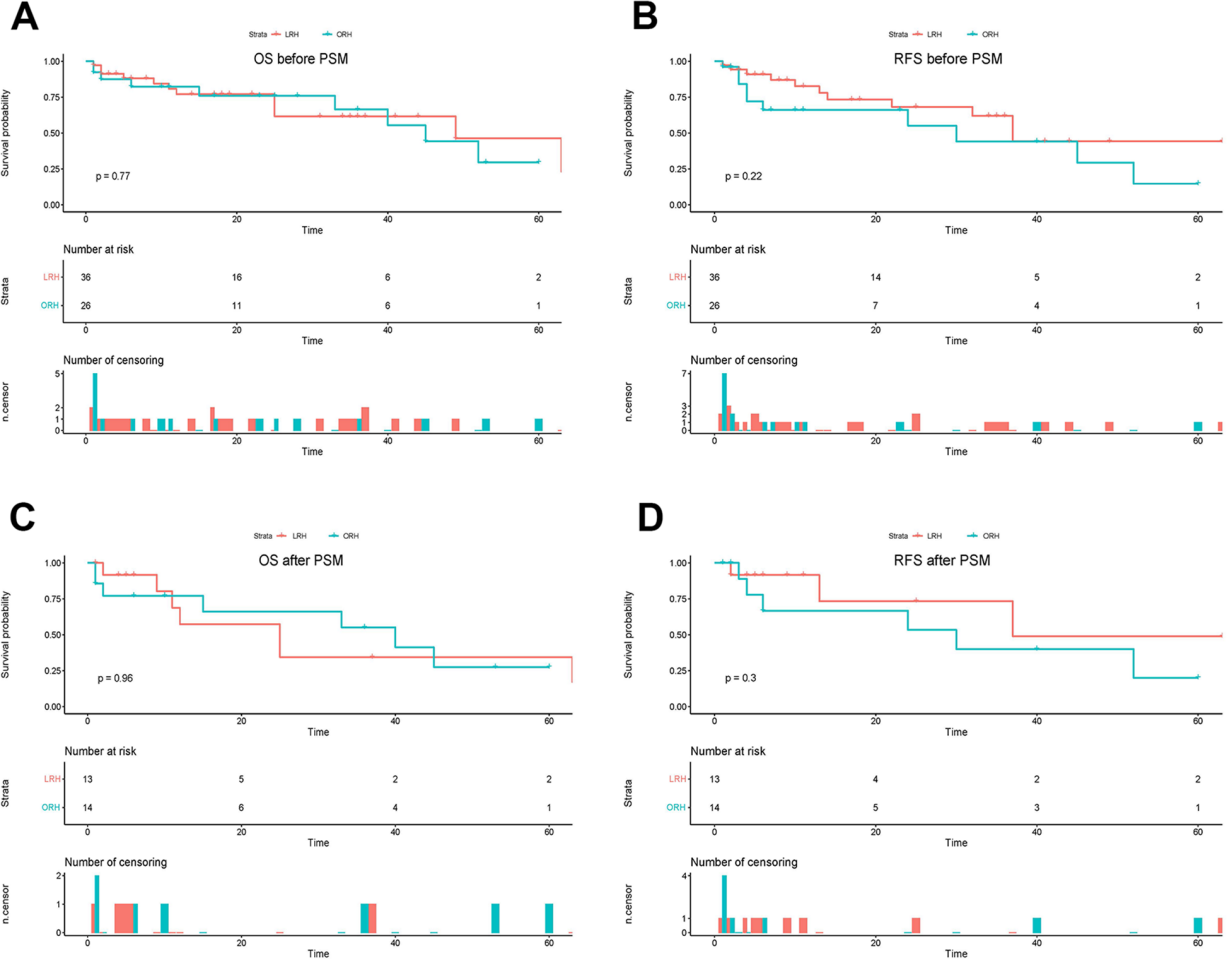
Comparison of overall survival (OS) and recurrence-free survival (RFS) rates between the laparoscopic repeat hepatectomy (LRH) and open repeat hepatectomy (ORH) groups. **A** Kaplan–Meier curve for OS in the overall cohort (*n* = 62). **B** Kaplan–Meier curve for RFS in the overall cohort (*n* = 62). **C** Kaplan–Meier curve for OS in the matched cohort (*n* = 27). **D** Kaplan–Meier curve for DFS in the matched cohort (*n* = 27)

The long-term outcomes in the entire and cohorts after matching were displayed in Fig. 2. The median follow-up time was 31.0 months (range: 1–65 months) in the total cohort and 37 months (range: 1–65 months) in the PSM cohort. The 1-, 3-, and 5-year recurrence-free survival (RFS) rates in the LRH group were 73.4%, 62.0%, and 44.3%, respectively; the corresponding rates in the ORH group were 66.1%, 44.1%, and 14.7%, respectively, (*p* = 0.22). The 1-, 3-, and 5-year overall survival (OS) rates of the LRH group were 77.1%, 61.6%, and 46.2%, respectively; the corresponding rates in the ORH group were 82.3%, 66.5%, and 29.5%, respectively (*p* = 0.77). Kaplan–Meier analysis indicated no statistically significant difference for OS and DFS between the two groups, and results remained similar in the PSM analysis (*p* = 0.96 and *p* = 0.30, respectively).

## Discussion

Hepatectomy has been considered to be an effective treatment for liver cancer patients with adequate liver remnant and preserved liver function. LH is now increasingly performed by many centers with expertise in both hepatectomy and advanced laparoscopy [14]. Compared with laparotomy, laparoscopic surgery has clearly shown the advantages of less intraoperative bleeding and postoperative pain, and shorter hospital stay, which is beneficial regarding long-term cancer prognosis [15–17]. Nevertheless, the cumulative recurrence percentage of postoperative HCC is as high as 70% at 5 years, and recurrence is one of the major causes of death in these patients [18]. Repeat hepatectomy is considered to be a viable therapy for patients with recurrent liver cancer [19]. However, repeat hepatectomy is more challenging because of the risk of intraoperative bleeding, biliary tract injury, and any other organ damage. The presence of server celiac adhesions, changes in anatomical positions, and impaired liver function brought by resection of the hepatic parenchyma with chronic liver diseases further increase the surgical complexity [20]. Extensive intra-abdominal adhesions have been considered a contraindication to laparoscopic repeat hepatectomy [3]. As liver cirrhosis, portal hypertension, and abdominal adhesions are common, the formation of collateral circulation in hepatic adhesions further increases surgical difficulty. Therefore, repeat hepatectomy requires detailed preoperative patient evaluation. Belli et al. [21] reported 15 recurrent HCC patients undergoing laparoscopic repeat hepatectomy and radiofrequency ablation. They concluded that a comprehensive preoperative evaluation is necessary and developed the inclusion criteria: Child-Pugh class A, tumor size < 5 cm, and tumors located in segments II–VI.

Owing to the developments in laparoscopic surgical experience, techniques, and instruments, the indications for LRH have expanded. Kanazawa et al. [16] reported 20 cases of LRH, with six tumors located in segments VII, VIII, and I, indicating that LRH can also be performed safely even for recurrent tumors located in difficult segments. Goh et al. [22]. retrospectively analyzed 103 patients with recurrent liver cancer undergoing laparoscopic surgery and demonstrated that LRH was of great efficacy for highly chosen patients, in centers with broad experience performing laparoscopic hepatectomy. LRH can also be performed in patients with background of previous open hepatectomy, previous major hepatectomy, previous multiple tumors, cirrhosis, ipsilateral HCC recurrence, and tumors located in difficult areas (e.g., right posterior lobe or caudate lobe). In our study, tumors measured < 5 cm in the LRH group, and 12 cases had tumors located in segments VII, VIII, and I. In our experience, LRH can be safely performed for relatively small lesions in all segments after careful judgement and procedure planning.

**Fig. 3:**
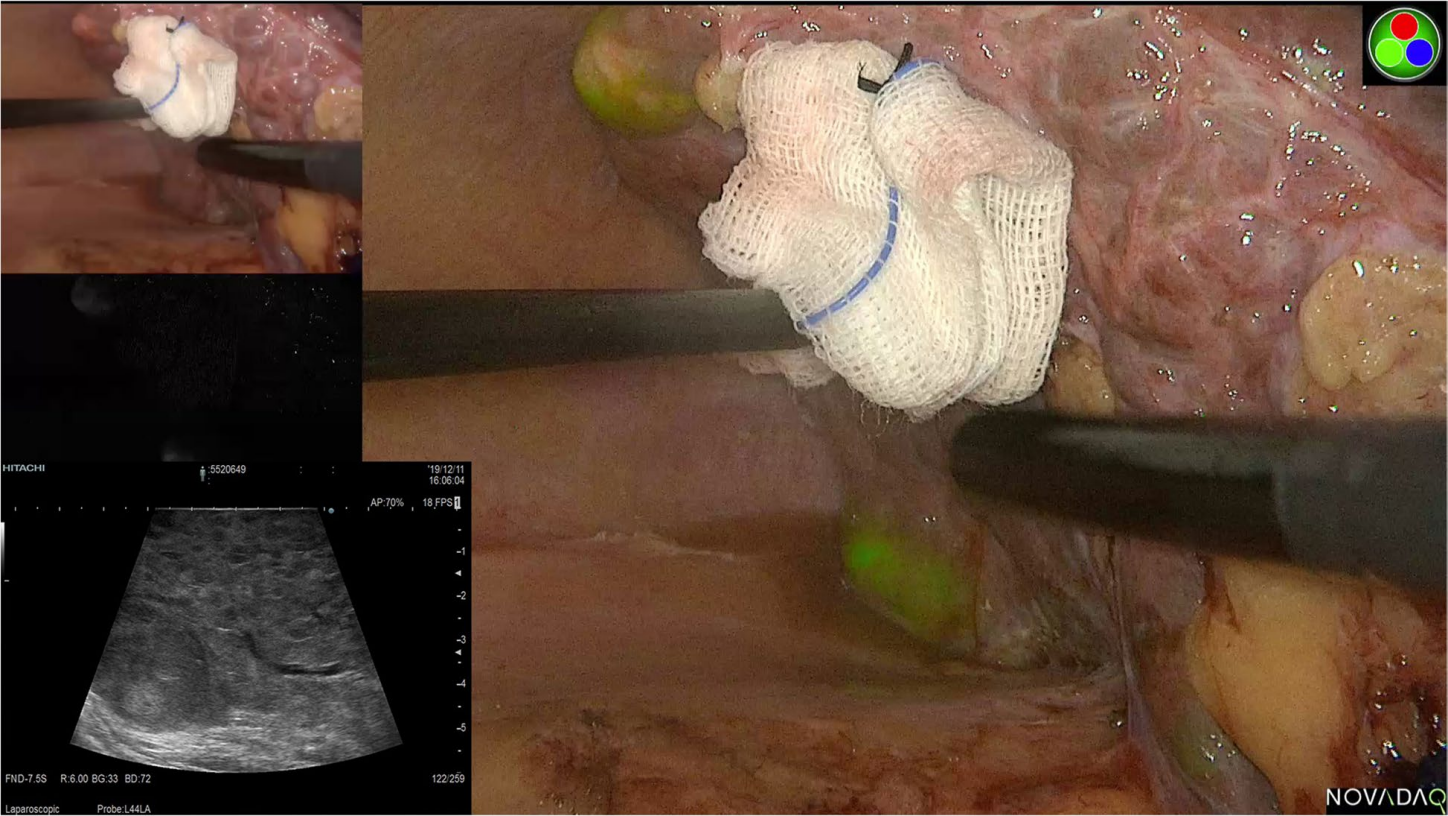
Laparoscopic ultrasound (LUS) combined with ICG fluorescence navigation to improve the intraoperative identification and demarcation of recurrent tumors

Owing to postoperative adhesions, and conversion in anatomical landmarks and liver deformity brought by the first hepatectomy, it is technically challenging to perform LRH for ipsilateral tumor recurrence, particularly for tumors in segments VII or VIII [23]. Indeed, LRH for neoplasms near the hepatic hilum and large vessels is technically difficult since it is hard to secure adequate surgical margins, even in the procedure of open repeat hepatectomy [24]. Furthermore, deficits in tactile sensation under laparoscopy could account for the difficulty defining the resection margin. Costal margins and diaphragmatic motion significantly restrict manipulation, which may result in insufficient tumor clearance [25, 26]. Precise localization of the tumor is crucial for a successful laparoscopic approach. In the present study, nine patients with tumors located in difficult liver segments (I, VII, and VIII) underwent complete R0 resection under purely laparoscopic surgery. In our experience, for deep lesions that are difficult to localize, we routinely use laparoscopic ultrasonography (LUS) intraoperatively to accurately determine tumor number and location and evaluate the adjacent relationships between the tumors and major intrahepatic vessels. Moreover, LUS can also be used to determine the hepatic plan of dissection to guide resection. Recently, indocyanine green (ICG) fluorescence navigation has been adopted for intraoperative visualization of HCC and other hepatic tumors [27]. Yoshioka et al. [28] reported a senior patient undergoing LRH with the guide of an ICG fluorescence navigation system, noting that ICG fluorescence navigation enabled clear intraoperative recognition of the tumor, even microscopic lesions that were not identified preoperatively. In our practice, we used LUS combined with ICG fluorescence navigation to improve the intraoperative identification and demarcation of tumors to facilitate complicated segmentectomy, which further decreased the number of surgical margin-positive patients (Fig. 3).

**Fig. 4:**
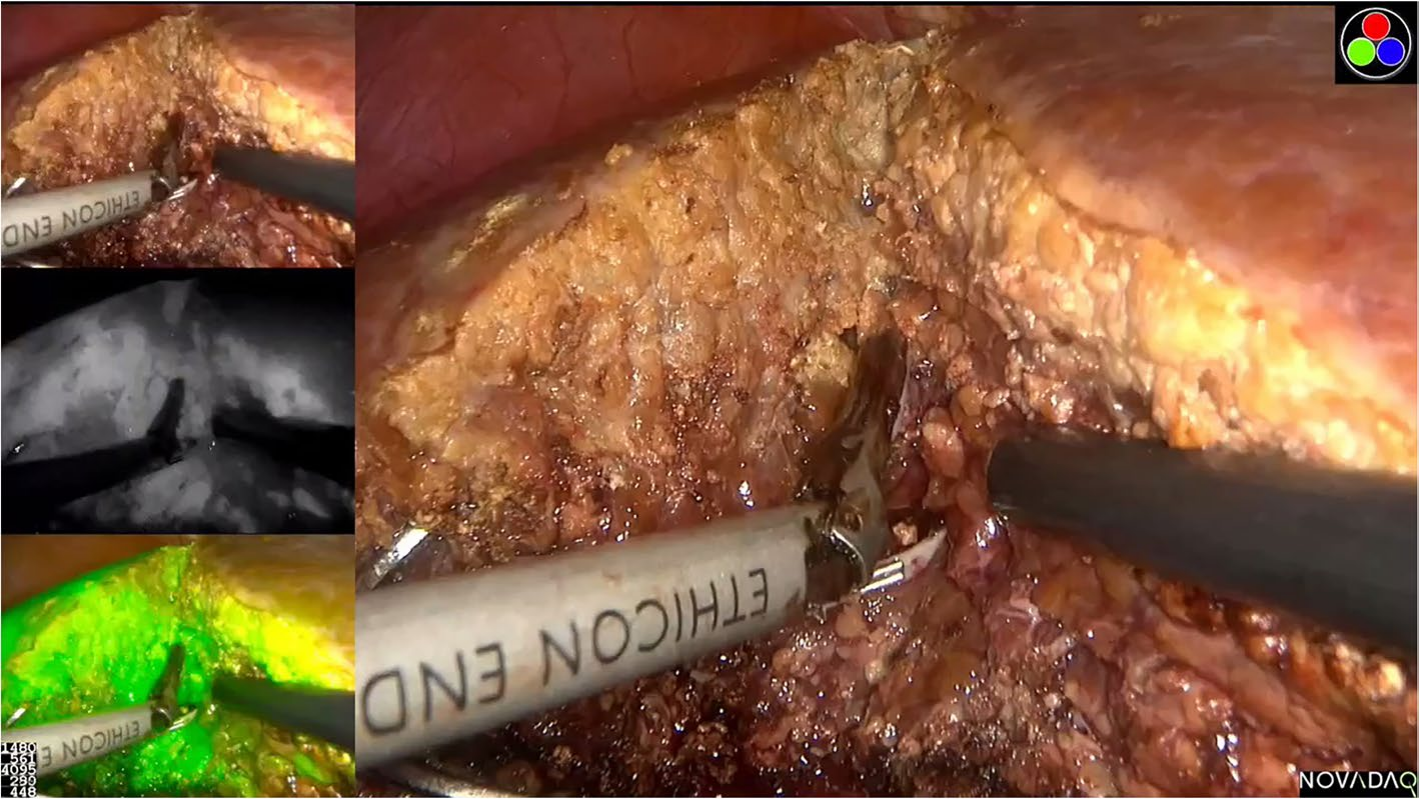
Two-hand technique of combining harmonic scalpel and laparoscopic Peng’s multifunction operative dissector (LPMOD) in recurrent HCC cases under ICG fluorescence background

Belli et al. [21] indicated that LRH was related with more limited operative time for patients with laparoscopic hepatectomy history. In contrast, Goh et al. [29] conducted a propensity score-matching study and demonstrated that LRH was associated with significantly longer operation times compared with ORH. In our series, operating time was comparable between the two groups after matching, and the surgical time for patients who had undergone previous laparoscopic hepatectomy was not less than that for patients who went through previous open hepatectomy. Noda et al. [30] analyzed the short-term postoperative outcomes of 20 LRH with 48 ORH cases and concluded that significantly less blood loss and lower occurrence of postoperative complications were seen in the LRH. In our study, we discovered that the estimated intraoperative blood loss of patients in the LRH group was significantly lower compared with the ORH group, which was consistent with the previous study. Furthermore, only one patient developed severe complications. The control of hepatic blood flow and procedure optimization when transecting the liver parenchyma remain essential to facilitate repeat hepatectomy and achieve less postoperative complications. In our institution, laparoscopic selective hemi-hepatic vascular occlusion is routinely performed in hemi-hepatectomy. This approach is considered safe and effective in hepatectomy because it has little effect on hepatic inflow to the remnant liver and prevents the liver from ischemia-reperfusion injury. In addition, we use the two-handed technique for major hepatectomy. Specifically, we used the LPMOD combined with the harmonic scalpel to transect the liver parenchyma. The LPMOD integrates functions, such as dissection, electrocoagulation, irrigation, and aspiration [31, 32]. We designed a novel two-handed technique that manage accidental hemorrhage shortly since it combines the small vessel sealing function of the harmonic scalpel with the hepatic parenchyma dissection function of the LPMOD (Fig. 4) [33].

A recent clinical study showed that the operation time was 279.3 ± 124.8 and 296.4 ± 155.3 for LRH and ORH, respectively, and there was no significant difference in operation time between LRH and ORH group (*P* = 0.6294) [34]. There was no significant difference in the operation time of the ORH group between our study and the existing literature. Still, the operation time of the LRH group before the PSM analysis was significantly less than that reported in the current literature. The reasons are as follows: First, the small sample size may lead to certain restrictions, and the conclusion needs to be further verified by a more extensive sample prospective study. Second, as a retrospective study, there may be some selection bias. For patients whose initial hepatectomy is laparoscopic hepatectomy, or open hepatectomy with relatively limited resection extent and acceptable abdominal adhesion condition judged by experience, surgeons often adopt LRH to remove recurrent lesions. Therefore, the LRH group may lack some patients whose initial operation is open hepatectomy with broad resection extent and severe abdominal adhesion conditions. Fortunately, there was no significant difference in the operation time between LRH and ORH groups, so such bias did not have a substantial impact on the final result. Third, with the improvement of technology and innovation of equipment, LRH also began to reveal its unique advantages. The enlargement of the operation area by laparoscopy and the tension formed by pneumoperitoneum are conducive to the detailed anatomy of the adhesion. Extensive adhesion release is not required since laparoscopic equipment can bypass some adhesions without separation and affecting operative field exposure [35, 36]. Laparoscopic operation is elaborate, which can minimize the movement of the liver and the damage to collateral circulation and lymphatic reflux in patients with liver cirrhosis. In addition, the conventional appliance of laparoscopic surgical equipment and technologies such as laparoscopic ICG fluorescence navigation technology, laparoscopic ultrasound, LPMOD, and laparoscopic regional blood flow blocking technology makes LRH more secure and efficient.

The first large propensity score-matching study of primary LH and OH for HCC has revealed that LH was related with less intraoperative bleeding, less morbidity and shorter length of hospitalization, but comparable long-term survival. According to Liu et al., the 1-year, 3-year, and 5-year disease-free survival rates were 79.0, 51.0, and 31.9%, respectively [37]. In our series, the median DFS of patients with recurrent HCC is 36 months, with 1-year, 3-year, 5-year RFS rates of 73.4, 62.0, and 44.3%, respectively. Encouraging results were observed in our series regarding the RFS were consistent with the previously reported series [36–38]. Therefore, it should never be neglected that the aggressive surgical intervention as the major treatment for the recurrence of the hepatic malignancies is associated with beneficial long-term survival, when implemented on selected patients.

The limitation of this study is associated with the existing potential heterogeneity of retrospective research. Furthermore, the sample size was still relatively small. Future multicenter researches with more sample capacity are required to obtain more comprehensive and accurate results for LRH. Furthermore, our study was performed under specific selection criteria, which may create potential selection bias. The years of cases included in this study are too long, and technical progress of open and laparoscopic hepatectomy may lead to the deviation of research results.

In conclusion, LRH is associated with less blood loss, lower blood transfusion rate, shorter hospital stay, and equally satisfactory oncological results compared to ORH. Compared with ORH, LRH also began to reveal its unique advantages with the improvement of technology and innovation of equipment. The enlargement of the operation area by laparoscopy and the tension formed by pneumoperitoneum are conducive to the detailed anatomy of the adhesion. Extensive adhesion release is not required since laparoscopic equipment can bypass some adhesions without separation and affecting operative field exposure [35, 36]. Laparoscopic operation is elaborate, which can minimize the movement of the liver and the damage to collateral circulation and lymphatic reflux in patients with liver cirrhosis. In addition, the conventional appliance of laparoscopic surgical equipment and technologies such as laparoscopic ICG fluorescence navigation technology, laparoscopic ultrasound, LPMOD, and laparoscopic regional blood flow blocking technology makes LRH more secure and efficient. Of course, it should be pointed out that LRH requires surgeons to experience a steep learning curve. In the future, related prospective research of large samples in this field also needs to be carried out in centers with rich laparoscopic surgical experience and technology.

## Data Availability

The datasets used and/or analyzed during the current study are available from the corresponding author on reasonable request.
